# Nonlinear heart rate variability biomarkers for gastric cancer severity: A pilot study

**DOI:** 10.1038/s41598-019-50358-y

**Published:** 2019-09-25

**Authors:** Bo Shi, Lili Wang, Chang Yan, Deli Chen, Mulin Liu, Peng Li

**Affiliations:** 1 School of Medical Imaging, Bengbu Medical College, Bengbu, Anhui 233030 China; 2 Department of Gastrointestinal Surgery, The First Affiliated Hospital of Bengbu Medical College, Bengbu, Anhui 233004 China; 3 School of Control Science and Engineering, Shandong University, Jinan, Shandong 250061 China; 4 Division of Sleep and Circadian Disorders, Brigham & Women’s Hospital, Harvard Medical School, Boston, 02115 MA USA

**Keywords:** Prognostic markers, Translational research

## Abstract

Identifying prognostic factors by affordable tools is crucial for guiding gastric cancer (GC) treatments especially at earlier stages for timing interventions. The autonomic function that is clinically assessed by heart rate variability (HRV) is involved in tumorigenesis. This pilot study was aimed to examine whether nonlinear indices of HRV can be biomarkers of GC severity. Sixty-one newly-diagnosed GC patients were enrolled. Presurgical serum fibrinogen (FIB), carcinoembryonic antigen (CEA), and carbohydrate antigen 19-9 (CA199) were examined. Resting electrocardiogram (ECG) of 5-min was collected prior to surgical treatments to enable the HRV analysis. Twelve nonlinear HRV indices covering the irregularity, complexity, asymmetry, and temporal correlation of heartbeat fluctuations were obtained. Increased short-range temporal correlations, decreased asymmetry, and increased irregularity of heartbeat fluctuations were associated with higher FIB level. Increased irregularity and decreased complexity were also associated with higher CEA level. These associations were independent of age, sex, BMI, alcohol consumption, history of diabetes, left ventricular ejection fraction, and anemia. The results support the hypothesis that perturbations in nonlinear dynamical patterns of HRV predict increased GC severity. Replication in larger samples as well as the examination of longitudinal associations of HRV nonlinear features with cancer prognosis/survival are warranted.

## Introduction

The fact of late-stage presentation and inaccessible treatment is urging an early diagnosis of cancer, the second leading cause of death worldwide. Such a malignancy spreads equally without preference on human beings all over the world but gastric cancer (GC) acts rather eccentrically that has been pushed out as an exception. It is so common in China and other East Asia countries as well, ranking the second in cancer death as opposed to the fifth globally^1^. To improve GC prognosis and facilitate a better treatment planning, early and sensitive diagnosis with feasible and affordable clinical measurements is essential.

Converging evidence has suggested a pivotal role of the autonomic control in tumor progression, in particular the contribution of the vagal nerve activity through many tumor-inhibiting mechanisms^2,3^. As a clinical routine, the measurement of electrocardiogram (ECG) or more specifically the analysis of the beat-to-beat ECG RR interval variations – the heart rate variability (HRV) – is an optimal noninvasive biomarker for the autonomic regulation^4,5^. In previous studies, reduced HRV was found in cancer patients compared to healthy peers^6^. Lower HRV at baseline was also reported to predict increased carcinoembryonic antigen (CEA) months later in a historical-prospective study^7^. In addition, in a most recent systematic review on HRV and cancer prognosis^8^, 19 studies that involved various kinds of cancer patients were included and appraised. Regardless of the cancer types, this review concluded an adverse effect of lower HRV towards shorter survival, higher tumor burden, or more advanced metastasis stage. Consistently, in another recent clinical study of GC patients^9^, lower HRV was found to be associated with advanced clinical stage, increased tumor size, tumor infiltration, lymph node metastasis, and involvement of distant organs.

Surprisingly, all studies reviewed above used only traditional linear HRV measures, albeit a commonly accepted nonlinear nature of HRV^10,11^. It is considered highly complex owning to the competition between spontaneity and adaptability of the heart beat regulation. Across a variety of studies in the field of cardiovascular diseases, nonlinear dynamical HRV analysis has shown a tremendous advantage over these linear time- and frequency-domain methods^10–17^. Therefore, we would like to examine the potential of nonlinear HRV measures in cancer diagnosis, prognosis, and treatment planning. At the time of analysis, 61 consecutive patients diagnosed with GC were enrolled. In this pilot phase, we explored the relationships of 12 commonly-used nonlinear HRV measures including (1) six entropy-based measures: approximate entropy (ApEn), sample entropy (SampEn), fuzzy entropy (FuzzyEn), permutation entropy (PermEn), conditional entropy (CE), distribution entropy (DistEn); (2) four asymmetry indices: Porta’s index (PI), Guzik’s index (GI), slope index (SI), area index (AI); and (3) detrended fluctuation analysis (DFA) derived metrics α_1_ and α_2_ with serum indices that are highly relevant to cancer prognosis including fibrinogen (FIB)^18,19^, CEA^20^, and carbohydrate antigen 19-9 (CA199)^21^. We hypothesized that patients with nonlinear HRV measures changing towards lower complexity/higher randomness had increased serum FIB, CEA, and CA199 levels.

## Results

**Figure 1:**
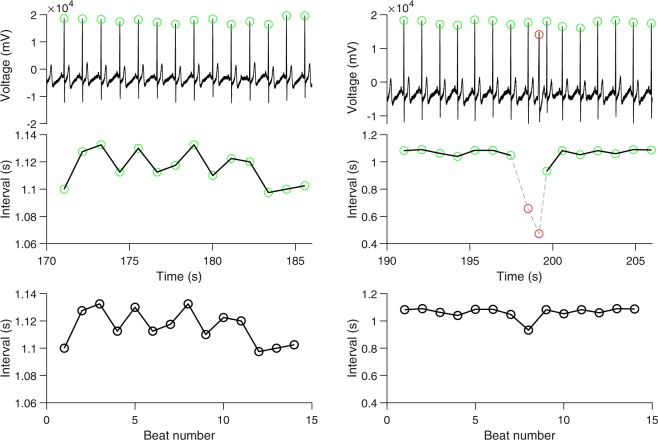
Construction of heart rate variability time-series. Upper panel: shown left an electrocardiogram (ECG) segment (a zoomed-in portion from the complete recording) without ectopic beats and right another ECG segment with ectopic beats (the beat marked in red). Middle panel: the time interval (RR interval) between the current R beat and the following R beat. Two anomaly intervals related to the ectopic beat are shown in red with gray dashed lines on the right-hand side. Bottom panel: the time-series used for analysis. The RR interval time-series on the left-hand side without anomalies is used directly for analysis. The anomalies on the right-hand side are removed and the resulted two pieces are sewed together to make one time-series for analysis.

Figure 1 shows examples of RR interval time-series that illustrate the construction of RR interval time-series from ECG without ectopic beats and with ectopic beats, respectively. Table 1 summarizes the demographics and the clinical and HRV measures of patients. Pearson correlation analyses resulted in six nonlinear HRV features, i.e., FuzzyEn, PermEn, DistEn, PI, α_1_, that had significant (*p* < 0.1) correlations with at least one of the three clinical GC parameters (Table 2). Based on these results, linear regression models of FIB with separately FuzzyEn, PermEn, PI, or α_1_ were performed; linear regression models of CEA with PermEn, DistEn, or PI separately were performed; linear regression models of CA199 with PI or GI were separately examined. Results from linear regressions were summarized in Table 3.

**Table 1:** Demographical, clinical, and HRV measures of patients.

Variables	Values
**Demographics**	
*N* (female/male)	61 (16/45)
Age (years)	63.6 (10.4)
BMI (kg/m^2^)	22.6 (3.3)
**Medical**	
History of alcohol consumption (yes/no)	9/52
History of diabetes (yes/no)	11/50
LVEF	56.9 (4.0)
**Hematology**	
FIB (g/L)	3.49 (0.84)
CEA	3.32 [4.66]
CA199	13.6 [48.31]
Hb	125.5 (21.4)
**HRV**	
ApEn	0.98 (0.14)
SampEn	1.82 (0.34)
FuzzyEn	1.35 (0.26)
PermEn	3.12 (0.25)
CE	1.95 (0.28)
DistEn	0.65 (0.08)
PI	0.51 (0.03)
GI	0.50 (0.01)
SI	0.50 (0.01)
AI	0.50 (0.01)
α_1_	1.13 (0.23)
α_2_	1.05 (0.19)

Values are expressed as mean (standard deviation) or median [inter-quartile range].

Abbreviations: ApEn = approximate entropy; AI = area index; BMI = body mass index; CA199 = carbohydrate antigen 19-9; CE = conditional entropy; CEA = carcinoembryonic antigen; DistEn = distribution entropy; FIB = fibrinogen; FuzzyEn = fuzzy entropy; GI = Guzik’s index; Hb = hemoglobin; LVEF = left ventricular ejection fraction; PermEn = permutation entropy; PI = Porta’s index; SampEn = sample entropy; SI = slope index.

**Table 2:** Bivariate correlation between clinical gastric cancer parameters and nonlinear HRV parameters.

	FIB	CEA	CA199
ApEn	(−0.09, 0.5)	(−0.05, 0.7)	(0.00, >0.9)
SampEn	(−0.09, 0.5)	(0.20, 0.1)	(0.00, >0.9)
FuzzyEn	**(−0**.**25**, **0**.**05)**	(0.05, 0.7)	(−0.01, >0.9)
PermEn	**(0**.**38**, **0**.**003)**	**(0**.**35**, **0**.**006)**	(0.03, 0.8)
CE	(−0.08, 0.5)	(0.05, 0.7)	(0.13, 0.3)
DistEn	(−0.21, 0.1)	**(−0**.**32**, **0**.**01)**	(0.05, 0.7)
PI	**(−0**.**44**, **0**.**0004)**	**(−0**.**26**, **0**.**05)**	**(−0**.**25**, **0**.**05)**
GI	(−0.20, 0.1)	(−0.06, 0.6)	**(−0**.**27**, **0**.**04)**
SI	(−0.17, 0.2)	(−0.15, 0.3)	(−0.20, 0.1)
AI	(−0.19, 0.2)	(0.04, 0.8)	(−0.19, 0.1)
α_1_	**(0**.**49**, <**0**.**0001)**	(−0.04, 0.8)	(0.05, 0.7)
α_2_	(0.21, 0.1)	(−0.05, 0.7)	(−0.07, 0.6)

Values are expressed as (*r*, *p*).

Bold indicates statistically significant at *p* < 0.1. Abbreviations: ApEn = approximate entropy; AI = area index; BMI = body mass index; CA199 = carbohydrate antigen 19-9; CE = conditional entropy; CEA = carcinoembryonic antigen; DistEn = distribution entropy; FIB = fibrinogen; FuzzyEn = fuzzy entropy; GI = Guzik’s index; PermEn = permutation entropy; PI = Porta’s index; SampEn = sample entropy; SI = slope index.

**Table 3:** Results from linear regression models (adjusted for age and sex).

Outcome	Predictor	Coefficient (Estimate ± SE)^*^	*p*	FDR-corrected *p*
FIB	α_1_	0.41 ± 0.10	0.0001	0.0009
FIB	PI	−0.35 ± 0.10	0.0009	0.004
FIB	PermEn	0.30 ± 0.11	0.007	0.02
CEA	PermEn	0.36 ± 0.15	0.02	0.04
CEA	DistEn	−0.32 ± 0.14	0.02	<0.05
CA199	GI	−0.65 ± 0.33	0.06	>0.05
CEA	PI	−0.27 ± 0.15	0.07	>0.05
CA199	PI	−0.44 ± 0.23	0.07	>0.05
FIB	FuzzyEn	−0.19 ± 0.11	0.08	>0.05

^*^Effects for 1-standard deviation increase in the predictor adjusted for covariates.

Abbreviations: CA199 = carbohydrate antigen 19-9; CEA = carcinoembryonic antigen; DistEn = distribution entropy; FDR: false discovery rate; FIB = fibrinogen; FuzzyEn = fuzzy entropy; GI = Guzik’s index; PermEn = permutation entropy; PI = Porta’s index; SE: standard error.

**Figure 2:**
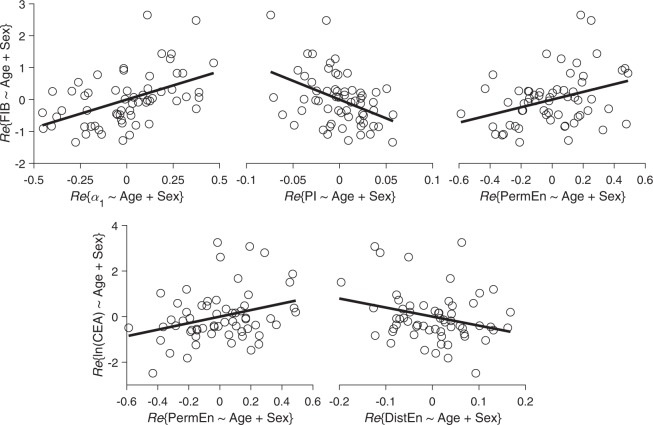
Partial correlation plots for the significant associations after correcting for FDR. *Re*{Y ~ X}: the residual for regressing Y against X. Abbreviations: CEA = carcinoembryonic antigen; DistEn = distribution entropy; FIB = fibrinogen; PermEn = permutation entropy; PI = Porta’s index.

After correcting for multiple comparisons, α_1_, PI, and PermEn were significantly associated with FIB, specifically, FIB increased by 0.41 ± 0.10, −0.35 ± 0.10, and 0.30 ± 0.11, respectively, for each 1-SD increase in α_1_, PI, and PermEn (all false discovery rate [FDR]-corrected *p* < 0.05). PermEn and DistEn were significantly associated with CEA, specifically, CEA increased by 0.36 ± 0.15 and −0.32 ± 0.14, respectively, for each 1-SD increase in PermEn and DistEn (both FDR-corrected *p* < 0.05). These five significant associations are further explained by the partial correlation plots as shown in Fig. 2 (the corresponding correlation plots without adjust for demographics were shown in Fig. S1 documented in the online Supplemental Materials). No parameters were significantly associated with CA199. After further adjusting for BMI, alcohol consumption, history of diabetes, Hb, and LVEF, all these associations still held with slightly changes in the estimated coefficients as shown in Table 4.

**Table 4:** Results from the augmented linear regression models (adjusted for age, sex, BMI, alcohol consumption, history of diabetes, Hb, and LVEF).

Outcome	Predictor	Coefficient (Estimate ± SE)^*^	*p*	FDR-corrected *p*
FIB	α_1_	0.41 ± 0.10	0.0002	0.002
FIB	PI	−0.34 ± 0.11	0.003	0.01
FIB	PermEn	0.33 ± 0.11	0.005	0.02
CEA	PermEn	0.38 ± 0.16	0.02	<0.05
CEA	DistEn	−0.34 ± 0.15	0.02	>0.05
CA199	PI	−0.41 ± 0.25	0.1	>0.05
FIB	FuzzyEn	−0.18 ± 0.12	>0.1	>0.05
CEA	PI	−0.24 ± 0.16	>0.1	>0.05
CA199	GI	−0.52 ± 0.37	>0.1	>0.05

^*^Effects for 1-standard deviation increase in the predictor adjusted for covariates.

Abbreviations: CA199 = carbohydrate antigen 19-9; CEA = carcinoembryonic antigen; DistEn = distribution entropy; FDR: false discovery rate; FIB = fibrinogen; FuzzyEn = fuzzy entropy; GI = Guzik’s index; Hb = hemoglobin; LVEF = left ventricular ejection fraction; PermEn = permutation entropy; PI = Porta’s index; SE: standard error.

The associations of FIB with α_1_, PI, and PermEn were also significant in secondary analysis using logistic regression with dichotomized outcomes, specifically, the odds of having higher FIB increased by 168% (95% confidence interval [CI]: [43%, 478%]), −52% (95% CI: [−76%, −14%]), and 79% (95% CI: [2%, 232%]), respectively, with each 1-SD increase in α_1_, PI, and PermEn (all *p* < 0.05; Table 5). The associations of CEA with PermEn and DistEn became not significant in the logistic regression models. However, PI showed significant associations with CA199 with an odds ratio of 0.53 (95% CI: [0.27, 0.95]) for 1-SD increase in PI (*p* = 0.03; Table 5).

**Table 5:** Results from Logistic regression models (adjusted for age and sex).

Outcome^a^	Predictor	OR (CI 95%)^*^	*p*
FIB	α_1_	2.68 (1.43, 5.78)	**0**.**001**
FIB	PI	0.48 (0.24, 0.86)	**0**.**01**
FIB	PermEn	1.79 (1.02, 3.32)	**0**.**04**
CEA	PermEn	1.62 (0.91, 3.03)	0.1
CEA	DistEn	0.61 (0.33, 1.06)	0.07
CA199	GI	0.45 (0.17, 1.06)	0.07
CEA	PI	0.64 (0.35, 1.14)	0.1
CA199	PI	0.53 (0.27, 0.95)	**0**.**03**
FIB	FuzzyEn	0.62 (0.34, 1.08)	0.09

Results presented in the same order as in Table 3. Bold *p* values indicate statistically significant at alpha = 0.05 level.

*Effects for 1-standard deviation increase in the predictor adjusted for covariates.

^a^Outcomes are each dichotomized with a threshold value: 3.5 for FIB, 5 for CEA, and 37 for CA199.

Abbreviations: CA199 = carbohydrate antigen 19-9; CEA = carcinoembryonic antigen; CI = confidence interval; DistEn = distribution entropy; FIB = fibrinogen; FuzzyEn = fuzzy entropy; GI = Guzik’s index; PermEn = permutation entropy; OR = odds ratio; PI = Porta’s index.

## Discussion

With 61 pathologically-diagnosed GC patients in this pilot study, for the first time we demonstrated significant associations between clinical cancer markers and several nonlinear HRV measures after accounting for multiple comparisons. Specifically, the increase of short-range temporal correlations in heartbeat fluctuations (i.e., increase in α_1_ which was calculated within time scales 4–16 beats), the decrease of the asymmetry in heartbeat acceleration/deceleration patterns (i.e., PI), and the increase of the irregularity of heartbeat fluctuations (i.e., PermEn) were associated with higher serum FIB level. The increase in PermEn as well as the decrease of the complexity of heartbeat dynamics (i.e., DistEn) were also associated with higher serum CEA level. Importantly, these associations were independent of several potential confounding factors including age, sex, BMI, alcohol consumption, history of diabetes, Hb, and LVEF.

### Nonlinear HRV measures as markers of autonomic nervous function modulating tumor progression

The autonomic nervous function is an accepted component involved in cancer etiology^22^. There is mounting evidence supporting the vagal neuromodulation hypothesis in tumorigenesis through its effects of anti-inflammation, antioxidative stress, and sympathetic activity^2,3,23^ including animal studies^24–26^ that demonstrated a causal pathway.

Through the analysis of vagal and sympathetic modulation on heartbeat, HRV is a well-known and widely-applied noninvasive tool for assessing the autonomic nervous function. Increased HRV has consistently been associated to better prognosis in cancer patients^7,8,22,27–30^. There is also an increasing preference in the biomedical science/engineering communities of using nonlinear analysis approaches as complements to the traditional linear methods. Coming from the fields of statistical physics and nonlinear dynamics, these nonlinear approaches could uniquely capture the information content (i.e., entropy-based measures), asymmetry, or scaling invariant properties (i.e., DFA), all of which has been shown to offer additional, valuable knowledge to the underlying control mechanism, i.e., the autonomic regulation^12,31–35^.

### Nonlinear HRV measures for the organisms’ plasticity and adaptability coping with stress

Although being not immediately interpretable with regards to the vagal or sympathetic regulation, there are published pilot studies that have already explored the effects of vagal and sympathetic outflows on several nonlinear measures of heartbeat dynamics including entropy measures and DFA scaling exponents^36,37^. Using both human and animal models, they have offered the direct evidence of autonomic control influencing the complex behavior of the heart.

A more traditional or systemic level viewpoint is that the nonlinear behavior of HRV is attributed to the competing regulation on the heart coming from the two branches of the autonomic nervous system and the spontaneity of the organism itself. Such competition renders healthy organisms high complexity, enabling daunting plasticity and adaptability to the stresses/perturbations of everyday life^38,39^. In parallel with this complex physiology hypothesis, aging and disease progressions are usually accompanied by a progressive reduction of the complexity^12,34,38,40^; and the other way around the degradation of the complexity also predicts future incidence of disease^41^.

In keeping with the complexity loss theory, our results suggest that GC patients with worse prognosis showed lower autonomic control complexity even though each different nonlinear metric showed different changing directions. Theoretically, the most complex system should be neither too random nor too regular; it should correspond to a critical point in-between^42^. The departure of α_1_ from the value 1, whichever direction, both imply a reduced complexity^43^. In our case, it was a reduction towards the regular side (i.e., increase of α_1_ towards 1.5). The decrease of PI indicates a loss of time irreversibility, an important property of complex system which suggests an evolution of the system to equilibrium or a loss of hysteresis^44^. The increase of PermEn indicates higher irregularity which suggests a seemingly controversial behavior as compared with α_1_. However, the calculation of PermEn focused on the fluctuation motifs composed of 3 heart beats that were not included in the calculation of α_1_ for sake of a robust fitting. The decrease of DistEn, although it is in nature an entropy metric, directly suggests a decrease in complexity as evidenced by the simulation analysis in the original DistEn study^45^.

### Potential clinical relevance and usefulness

With the rising of global GC epidemic, the assessment of HRV may potentially meet the clinical urgency in three ways:

1. HRV analysis may offer a sensitive and noninvasive tool targeting an early GC diagnosis. On one hand, the nonlinear approaches used in this study are necessary in the way that they cope well with the nonlinear and nonstationary nature, resulting thus in a more robust assessment as compared with the existing linear methods. On the other hand, it might be possible to leverage these nonlinear indices with the existing linear measures to construct an integrated biomarker for early GC diagnosis. Further studies with larger samples are required to test this hypothesis.
2. HRV could help with the evaluation of prognosis and treatment planning for GC patients. The link between HRV and survival time suggests a role of HRV in helping screen the general health status and prognosis of cancer patients^8,29,30^. Previous studies also discovered a link between serum FIB and adjacent organ involvement^18^. Given the strong associations of serum FIB with HRV nonlinear indices reported in the current study, HRV analysis may thrive as a sensitive tool for the surgical planning of GC patients. Further clinicopathological studies are warranted to formally examine their associations.
3. HRV might be a target for interventions to prevent the disease or to slow down the progression. Although only cross-sectional associations were reported in this current study, previous animal studies have established a causal link between vagal activity and tumor genesis^24–26^. A case-control study that focuses on exposure to interventions improving HRV especially in terms of nonlinear properties is required to validate this causal pathway in humans.

### Study limitations

There are several notable limitations. First, the sample size is relatively small. Aside from the four indices (i.e., α_1_, PI, PermEn, DistEn) the other nonlinear HRV measures may also be correlated with the serum markers while their negative observations may simply be due to the power issue. Second, the cross-sectional nature of the study design limits our inference about longitudinal prediction ability of HRV nonlinear measures. It is of great clinical value to examine whether those presurgical HRV indices can predict longer term outcomes including treatment response and survival. Besides, it is also meaningful to check whether degradations of these nonlinear properties in otherwise normal people predict higher risk of developing GC later. Third, although the FIB, CEA, and CA199 are well developed serum markers of cancer severity or prognosis, the gold standard is pathological examination. We are still working to retrieve and pool detailed pathology data together in addition to the final diagnosis and are expecting to scrutinize their interrelationships with HRV nonlinear features in follow-up studies.

## Materials and Methods

### Patients and data collection

This study was approved by the Institutional Review Board of The First Affiliated Hospital of Bengbu Medical College and was performed in accordance with the ethical standards laid down in the 1964 Declaration of Helsinki and its later amendments. From March 2018 to December 2018, 126 patients were diagnosed with GC based on endoscopy and pathological examinations in The First Affiliated Hospital of Bengbu Medical College. Among them, 90 patients provided written informed consent and were enrolled in this study.

Serum FIB, CEA, and CA199 levels were examined before breakfast 1-week before surgical treatments. FIB levels were determined using the Clauss method (Sysmex CS51000, Sysmex Corporation, Kobe, Japan). Chemiluminescent assays were used to determine the CEA and CA199 levels (Architect i2000sr, Abbott Diagnostics, Abbott Park, IL, USA). ECG data were recorded continuously for 5 min one day before treatments with patients lying down for at least 20 min before the formal collection (HeaLink-R211B, HeaLink Ltd., Bengbu, China). The sampling frequency of ECG collection was 400 Hz. The precordial V5 lead was configured and the Ag/AgCl disposable electrodes were used (Junkang Ltd., Shanghai, China).

We further excluded participants with the following conditions: (1) recurrent GC (*N* = 1), (2) poor ECG quality (*N* = 2), (3) presence of ectopic beats (>10% of all beats; *N* = 8), or (4) administering of blood transfusion (*N* = 15) or chemotherapy (N = 3) prior to ECG collection. Therefore, data of 61 participants were presented and analyzed in this current study. Their demographics and clinical characteristics were shown in Table 1.

### Nonlinear HRV analysis

R-wave peaks were located automatically using a template-matching approach^46^ followed by visual inspections. Ectopic beats were identified manually during the visual inspection (1–3 ectopic beats, either premature atrial contraction or premature ventricular contraction, presented in 14 out of the 61 patients). The final HRV time-series were constructed for each patient by consecutive normal sinus R-R intervals. The normal to ectopic or ectopic to normal intervals were discarded and the corresponding two segments were stitched together to assure a reasonable length of the RR interval time-series. Figure 1 shows examples of the construction of RR interval time-series without and with ectopic beats.

For each RR interval time-series, 12 nonlinear HRV indices covering the irregularity, complexity, asymmetry, and temporal correlation of heartbeat fluctuations were calculated. The 12 indices included (1) six entropy-based measures: approximate entropy (ApEn), sample entropy (SampEn), fuzzy entropy (FuzzyEn), permutation entropy (PermEn), conditional entropy (CE), distribution entropy (DistEn); (2) four asymmetry indices: Porta’s index (PI), Guzik’s index (GI), slope index (SI), area index (AI); and (3) two detrended fluctuation analysis (DFA) derived metrics α_1_ and α_2_. The detail algorithms for calculating these indices were summarized in Supplemental Methods documented in the online Supplemental Materials.

The extraction of R-peaks, visual inspections of ectopic beats, and asymmetry analysis were done in MATLAB (Ver. R2018a, The MathWorks Inc., Natick, MA, US). Entropy analysis was performed using the EZ Entropy software^47^. The Kubios HRV software was used to perform the DFA^48^.

### Statistical analysis

Histograms of CEA and CA199 both showed an obvious right skewness; they were thus natural-log-transformed prior to further analysis (unless otherwise indicated). Bivariate Pearson correlations of FIB, CEA, and CA199 with each of the 12 nonlinear HRV measures were performed to screen potential predictors. Conservatively, features with *p* level of <0.1 were considered significant. Linear regressions were then performed for these pairs that passed this screening process with either FIB, CEA, or CA199 as an outcome. These models were adjusted for age and sex. To avoid collinearity, the HRV features were each included in a separate regression model as a predictor. To determine the statistical significance, the Benjamini-Hochberg procedure was used to control for false discovery rate (FDR) within multiple comparisons^49^. FDR-corrected *p* < 0.05 is considered statistically significant. These models were then augmented by further adjusting for BMI^50^, alcohol consumption^51^, history of diabetes^52^, LVEF^53^, and anemia as assessed by Hb^54^ followed by the same multiple comparison correction procedure. As an exploratory analysis, FIB, CEA, and CA199 were dichotomized each by a separate threshold value^19^: (1) FIB was considered high if >3.5 mg/mL and low if otherwise; (2) CEA was considered high if the original CEA level ≥ 5 and low if otherwise; and (3) CA199 was considered high if the original CA199 level ≥ 37 and low if otherwise. Logistic regression models were performed with each of the three dichotomized clinical parameters as an outcome and with each feature in the corresponding significant HRV feature set as a predictor while adjusted for age and sex. All statistical analyses were performed using the JMP Pro (ver. 14, SAS Institute, Cary, NC).

## Supplementary information

Supplemental Materials

## Data Availability

The datasets generated during and/or analysed during the current study are available from the corresponding author on reasonable request.
