# Supplementary material for: Nonlinear heart rate variability biomarkers for gastric cancer severity: A pilot study
Source: Sci Rep. 2019 Sep 25;9:13833. doi: 10.1038/s41598-019-50358-y (PMC6761171; doi:10.1038/s41598-019-50358-y)
Supplement: Supplementary file 1 — Supplemental Materials [file 41598_2019_50358_MOESM1_ESM.docx]

Nonlinear heart rate variability biomarkers for gastric cancer severity: A pilot study

Bo Shi^1^, Lili Wang^2^, Chang Yan^3^, Deli Chen^2^, Mulin Liu^2^, Peng Li^3,4,*^

^1^School of Medical Imaging, Bengbu Medical College, Bengbu, Anhui 233030, China

^2^Department of Gastrointestinal Surgery, The First Affiliated Hospital of Bengbu Medical College, Bengbu, Anhui 233004, China

^3^School of Control Science and Engineering, Shandong University, Jinan, Shandong 250061, China

^4^Division of Sleep and Circadian Disorders, Brigham & Women’s Hospital, Harvard Medical

*** Correspondence:**Peng Li
[pli9@bwh.harvard.edu](mailto:pli9@bwh.harvard.edu)

# Supplemental Methods

## Nonlinear HRV analysis

### Entropy analysis

For an HRV time-series $\mathbf{x}=x(i),1\leq i\leq N,$ its *m*-dimensional state space reconstruction can be obtained by

$u_{m}(i)=\{x(i),x(i+\tau),...,x(i+(m-1)\tau)\},$ (1)

where $1\leq i\leq N-m\tau$; $\tau$ and *m* represent the time delay parameter and the dimension parameter, respectively. The entropy-based measures focus on how fast new information as characterized by vectors or motifs is generated or how information is distributed in the state space representation (see ^1^ for a review of the entropy metrics for physiological time-series analysis). The information is rendered new if a new vector or signal motif presents – a vector that is dissimilar with previous vectors. To quantify whether two vectors are similar, the Chebyshev distance between them is usually used which can be calculated by

$d\left[ u_{m}(i),u_{m}(j) \right]=\max_{0\leq k\leq m-1}\left( \left| x(i+k\tau)-x(j+k\tau) \right| \right),1\leq i,j\leq N-m\tau,$ (2)

(1) Approximate entropy (ApEn). The percentage of the vectors $u_{m}(j)$ that are within *r* of $u_{m}(i)$ is calculated by

$C_{i}^{(m)}(r)=\frac{N_{i}^{(m)}(r)}{N-m\tau},$ (3)

where, $N_{i}^{(m)}(r)$ indicates the number of *j*’s that meet $d_{i,j}\leq r$, and $1\leq j\leq N-m\tau$. The mean of $C_{i}^{(m)}(r)$ over $1\leq i\leq N-m\tau$, after logarithmic transform is defined by $\Phi^{(m)}(r)=\frac{1}{N-m\tau}\sum_{i=1}^{N-m\tau} {\ln C}_{i}^{(m)} (r)$. In a similar way, $\Phi^{(m+1)}(r)$ is defined after increasing the dimension to *m*+1. The ApEn value of the time-series $\mathbf{x}$ can be calculated by ^2^

$ApEn(m,\tau,r)=\Phi^{(m)}(r)-\Phi^{(m+1)}(r)$. (4)

(2) Sample entropy (SampEn). Define $A_{i}^{(m)}(r)=\frac{N_{i}^{(m)}(r)}{N-m\tau-1}$ as the percentage of the vectors $u_{m}(j)$ that are within *r* of $u_{m}(i)$, where $N_{i}^{(m)}(r)$ indicates the number of *j*’s that meet $d_{i,j}\leq r$, and $1\leq j\leq N-m\tau,j\neq i$ to exclude self-matches. Denote $\Psi^{(m)}(r)=\frac{1}{N-m\tau}\sum_{i=1}^{N-m\tau} A_{i}^{(m)}(r)$ as the average of the percentage $A_{i}^{(m)}(r)$ over $1\leq i\leq N-m\tau$. In a similar way $\Psi^{(m+1)}(r)$ can be defined after increasing the dimension to *m*+1. The SampEn value of the time-series $\mathbf{x}$ can be calculated by ^3^

$SampEn(m,\tau,r)=-\ln\frac{\Psi^{(m+1)}(r)}{\Psi^{(m)}(r)}$. (5)

(3) Fuzzy entropy (FuzzyEn). Methodologically, FuzzyEn is quite similar as SampEn except that it replaces the percentage of vectors $u_{m}(j)$ within *r* of $u_{m}(i)$ with the average degree of membership which offers reliability especially for short-length data. For a given fuzzy membership function $e^{-\ln(2)(x/y)^{2}}$, $A_{i}^{(m)}=\frac{\sum_{j=1,j\neq i}^{N-m\tau} e^{-\ln(2)(x/y)^{2}}}{N-m\tau-1}$ is used ^1,4^ and, then, $\Psi^{(m)}(r)$ can be defined in similar ways as done in SampEn. FuzzyEn can be obtained by plugging them into equation (5).

(4) Permutation entropy (PermEn). PermEn evaluates the complexity by mapping the time series into a symbolic sequence. It ranks the state-space vectors in ascending order to obtain a permutation vector **π**. Note that the ranks of two equal values are deﬁned according to the orders of appearance. Here, we denote the frequency of each element $\pi_{j},1\leq j\leq m!$ as $p_{j}(m,\tau)$. Then, the PermEn can be calculated by ^1,5,6^

$PermEn(m,\tau)=-\frac{1}{{log}_{2} m!}\sum_{j=1}^{m!} p_{j}(m,\tau)\log_{2} \left[ p_{j}(m,\tau) \right]$ (6)

(5) Conditional entropy (CE). The full range of $\mathbf{x}$ is divided into a ﬁxed number of $\xi$ values labelled from zero to $\xi-1$. The coarse-graining resolution thus equals $\left[ \max(x)-\min(u) \right]/\xi$. It renders $x(i)$ sequences of symbols $\hat{x}(i),i=1,2,...,N$. Here $\xi$ indicates the quantization level. Define $u_{m}(i)$ and $u_{m}(j)$ by:

$\begin{matrix} u_{m}(i)=\left[ \hat{x}(i),\hat{x}(i-\tau),...\hat{x}(i-(m-1)\tau) \right] \\ u_{m+1}(j)=\left[ \hat{x}(j),u_{m}(j-\tau) \right] \end{matrix},$ (7)

respectively, where $(m-1)\tau+1\leq i,j\leq N$. The vectors $u_{m}(i)$ and $u_{m}(j)$ can be codified in decimal format as:

$\begin{matrix} \{u_{m}(i){\}}_{10}=\hat{x}(i)\xi^{m-1}+\hat{x}(i-\tau)\xi^{m-2}+...+\hat{x}(i-(m-1)\tau)\xi^{0}=w_{i} \\ \{u_{m+1}(i){\}}_{10}=\hat{x}(j)\xi^{m}+\{u_{m}(j-\tau){\}}_{10}=z_{i} \end{matrix},$ (8)

thus rendering each sequence of vectors $u_{m}(i)$ and $u_{m}(j)$ series of integer numers $w_{i}$ and $z_{j}$ with $w_{i}$ ranging from zero to $(\xi-1)\sum_{i=1}^{m-1} \xi^{i}$, and $z_{j}$ from zero to $(\xi-1)\sum_{j=1}^{m} \xi^{j}$. Deﬁne CE by ^1,6,7^

$CE(m,\tau)=SE(z_{j})-SE(w_{i})+perc(m)SE(1)$, (9)

where $SE(\cdot)$calculates the Shannon entropy of a specific distribution, $perc(m)$ is the percentage of $w_{i}$ patters found only once in the data set, $SE(1)$ the Shannon entropy of the quantized series $\hat{x}(i)$.

(6) Distribution entropy (DistEn). Instead of only calculating the probability of similar vectors, DistEn quantifies the complete information of the matrix $d_{i,j}\leq r,j\leq N-m\tau$ by estimating the Shannon entropy of all distances. Specifically, a histogram approach with a fixed bin number *B* is used to estimate the empirical probability density function of the distance matrix $d_{i,j}\leq r$ except the main diagonal (i.e., $i\neq j$). Using $\{p_{t},t=1,2,...,B\}$ to denote the probability of each bin, DistEn can be defined by the following formula ^8^

$DistEn(m,\tau,B)=-\frac{1}{{log}_{2} (B)}\sum_{t=1}^{B} p_{t}{log}_{2} (p_{t})$. (10)

Generally, the ApEn, SampE, FuzzyEn, PermEn, and CE are accepted to be direct measures of the irregularity, while the DistEn is sensitive to change of the complexity of time-series. For these entropy analyses, the following assignments of parameters were used based on previous studies: the time delay $\tau=1$, the embedding dimension $m=2$, the threshold value $r=0.25$, the quantification level $\xi=6$, and the bin number $B=256$.

### Asymmetry analysis

The following four well-studied asymmetry metrics derived from the Poincaré plot were used to estimate the asymmetry of heartbeat fluctuations ^9^. Larger values of these indices render increased asymmetry.

(1) Porta’s index (PI). Conceptually, PI renders symmetry when the numbers of points in the two regions in a Poincaré plot separated by the line of identity (LI) are the same and renders asymmetry if they differ ^10^. Thus, PI can be calculated by

$PI=\frac{a}{m}\times100$, (11)

wherein, $a$ is the number of points above the LI and $m$ the total number of points (points on the LI excluded).

(2) Guzik’s index (GI). GI uses the distances of points to the LI as a measure to assess whether the contributions of points in the two different regions in Poincaré plot are equal or not ^11^. Specifically,

$GI=\frac{\sum_{i=1}^{a} D_{i}}{\sum_{i=1}^{m} D_{i}}\times100$, (12)

wherein, $D_{i}$ is the Euclidian distance of point $i$ to the LI, i.e., $D_{i}=\frac{\left| x\left( i+1 \right)-x(i) \right|}{\sqrt{2}}$.

(3) Slope index (SI). The average phase angles of points in the two different regions in a Poincaré plot are calculated and used to assess the asymmetry ^12^. Specifically,

$SI=\frac{\sum_{i=1}^{a} \left| R\theta_{i} \right|}{\sum_{i=1}^{m} \left| R\theta_{i} \right|}\times100$, (13)

wherein, ${R\theta}_{i}=\frac{\pi}{4}-\theta_{i}$. $\theta_{i}=\mathrm{atan}\left( \frac{x(i+1)}{x(i)} \right)$ is the phase angle of point $i$; $\frac{\pi}{4}$ is the phase angle of the LI, i.e., $\mathrm{atan}\left( 1 \right)$.

(4) Area asymmetry (AI). The average areas of sectors formed by the points and the LI are calculated and used to assess the asymmetry ^13^. Specifically,

$AI=\frac{\sum_{i=1}^{a} S_{i}}{\sum_{i=1}^{m} S_{i}}\times100$, (14)

wherein, $S_{i}=\frac{1}{2}{\times R\theta}_{i}\times r^{2}$ is the area of the sector formed by point $i$ and LI; $r$ is the radius of the sector.

### Detrended fluctuation analysis (DFA)

DFA examines the temporal correlations of RR intervals at multiple time scales. Specifically, it first removes the global mean and integrates the time-series, i.e., $X_{t}=\sum_{i=1}^{t} \left( x(i)-\bar{x} \right)$ where $\bar{x}$ denotes the mean value of the time-series $x(i)$. The second step is to divide the integrated signal into non-overlapping windows of length *n* and to remove the trend of the integrated signal in each window using polynomial functions to obtain residuals, i.e., $\hat{X}_{t}=X_{t}-Y_{t}$ where $Y_{t}$ denotes the trend obtained by polynomial fit and $\hat{X}_{t}$ the integrated time series after detrending. It then calculates the root mean square of the residuals across all windows, obtaining the detrended fluctuation amplitude F(*n*), i.e., $F\left( n \right)=\sqrt{\frac{1}{N}\sum_{t=1}^{N} {\hat{X}_{t}}^{2}}$. The same steps are repeated for different window lengths, or time scales *n*. For a self-similar (or fractal) process, there usually exists a power-law form of F(*n*) in relation to $n$, i.e., F(*n*)~*n^α^*. The parameter *α*, called the scaling exponent, quantifies the temporal correlation as follows: if *α* = 0.5, there is no correlation in the fluctuations (“white noise”); if *α* > 0.5, there are positive correlations, where large values are more likely to be followed by large values (and vice versa); if *α* < 0.5, there are negative correlations, where large values are more likely to be followed by small values (and vice versa). The *α* values that are close to 1.0 have been observed in many physiological outputs under healthy young conditions ^14–17^, indicating the most complex underlying control mechanisms. The parameter *α* was fitted at two different time scale regions, resulting in two metrics: (1) *α*_1_ which is for *n* between 4-16 beats and (2) *α*_2_ which is for *n* between 16-64 beats ^18^. We note that in general, a longer recording is required to have more robust estimation of α_2_. Our selection of these two time scale regions was actually based upon the original paper talking about the cross-over phenomenon of the fluctuation function ^19^ as a reasonable tradeoff in clinical settings where usually short-term electrocardiogram measurements are usually performed. Besides, the two ranges have the same length on a log scale (i.e., $\log16-\log4=\log64-\log16$) so that on the $\log F\left( n \right)\sim\log n$ plane, a linear fitting within the range of $\left[ \log4,\log16 \right]$ generally possesses the same precision/accuracy as a linear fitting within the range of $\left[ \log16,\log64 \right]$ in terms of the support length.

# Supplemental Figures


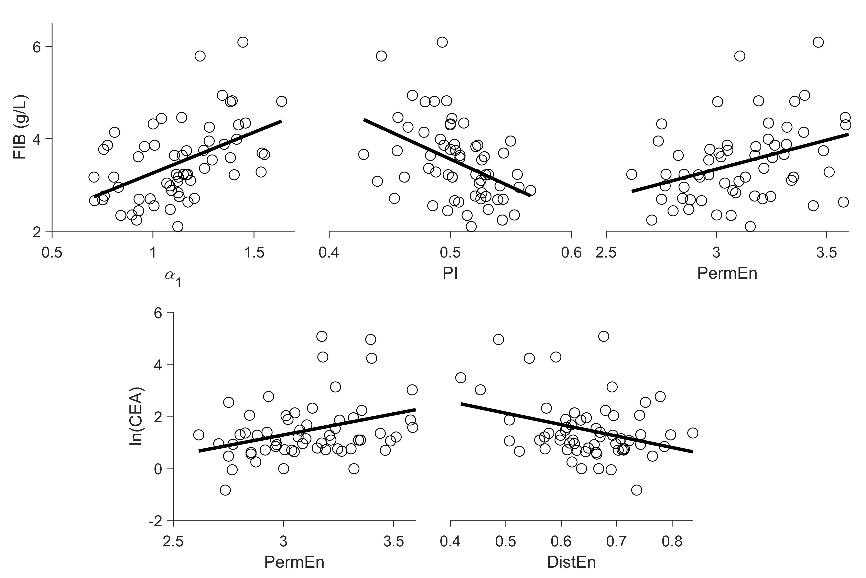


**Figure S1**. The correlation plots corresponding to Fig. 1 on the original scale without adjusting for demographics.

# References

1. Li, P. EZ Entropy: a software application for the entropy analysis of physiological time-series. *BioMedical Engineering OnLine* **18**, 30 (2019).

2. Pincus, S. Approximate entropy (ApEn) as a complexity measure. *Chaos* **5**, 110–117 (1995).

3. Richman, J. S. & Moorman, J. R. Physiological time-series analysis using approximate entropy and sample entropy. *American Journal of Physiology - Heart and Circulatory Physiology* **278**, H2039–H2049 (2000).

4. Chen, W., Zhuang, J., Yu, W. & Wang, Z. Measuring complexity using FuzzyEn, ApEn, and SampEn. *Med Eng Phys* **31**, 61–68 (2009).

5. Bandt, C. & Pompe, B. Permutation Entropy: A Natural Complexity Measure for Time Series. *Phys. Rev. Lett.* **88**, 174102 (2002).

6. Bo Shi, Yudong Zhang, Chaochao Yuan, Shuihua Wang & Peng Li. Entropy Analysis of Short-Term Heartbeat Interval Time Series during Regular Walking. *Entropy* **19**, 568 (2017).

7. Porta, A. *et al.* Measuring regularity by means of a corrected conditional entropy in sympathetic outflow. *Biol Cybern* **78**, 71–78 (1998).

8. Li, P. *et al.* Assessing the complexity of short-term heartbeat interval series by distribution entropy. *Med Biol Eng Comput* **53**, 77–87 (2015).

9. Wang, X. *et al.* Does the Temporal Asymmetry of Short-Term Heart Rate Variability Change during Regular Walking? A Pilot Study of Healthy Young Subjects. *Computational and Mathematical Methods in Medicine* **2018**, 1–9 (2018).

10. Porta, A., D’addio, G., Bassani, T., Maestri, R. & Pinna, G. D. Assessment of cardiovascular regulation through irreversibility analysis of heart period variability: a 24 hours Holter study in healthy and chronic heart failure populations. *Philos Trans A Math Phys Eng Sci* **367**, 1359–1375 (2009).

11. Guzik, P., Piskorski, J., Krauze, T., Wykretowicz, A. & Wysocki, H. Heart rate asymmetry by Poincaré plots of RR intervals. *Biomed Tech (Berl)* **51**, 272–275 (2006).

12. Karmakar, C. K., Khandoker, A. H. & Palaniswami, M. Phase asymmetry of heart rate variability signal. *Physiol Meas* **36**, 303–314 (2015).

13. Yan, C. *et al.* Area asymmetry of heart rate variability signal. *Biomed Eng Online* **16**, 112 (2017).

14. Peng, C. K. *et al.* Fractal mechanisms and heart rate dynamics. Long-range correlations and their breakdown with disease. *J Electrocardiol* **28 Suppl**, 59–65 (1995).

15. Peng, C. K. *et al.* Quantifying fractal dynamics of human respiration: age and gender effects. *Ann Biomed Eng* **30**, 683–692 (2002).

16. Hausdorff, J. M. *et al.* Altered fractal dynamics of gait: reduced stride-interval correlations with aging and Huntington’s disease. *J. Appl. Physiol.* **82**, 262–269 (1997).

17. Hu, K. *et al.* Non-random fluctuations and multi-scale dynamics regulation of human activity. *Physica A* **337**, 307–318 (2004).

18. Tarvainen, M. P., Niskanen, J.-P., Lipponen, J. A., Ranta-aho, P. O. & Karjalainen, P. A. Kubios HRV – Heart rate variability analysis software. *Computer Methods and Programs in Biomedicine* **113**, 210–220 (2014).

19. Peng, C. K., Havlin, S., Stanley, H. E. & Goldberger, A. L. Quantification of scaling exponents and crossover phenomena in nonstationary heartbeat time series. *Chaos* **5**, 82–87 (1995).
